# Exoscope‐Assisted Ovarian‐Sparing Resection of Contralateral Mature Teratomas in Bilateral Pediatric Ovarian Disease: A Case Series

**DOI:** 10.1111/ases.70331

**Published:** 2026-06-18

**Authors:** Wilson E. de Oliveira, Bianca Rezende Rosa, Gisele Eiras Martins, Gustavo da Silveira Orsi, Letícia Semissato dos Santos, Ana Laura Balduino do Nascimento, Luiz Fernando Lopes, Rodrigo Chaves Ribeiro

**Affiliations:** ^1^ Department of Pediatric Surgery Children's Cancer Hospital, Barretos Cancer Hospital Barretos São Paulo Brazil; ^2^ “Innovation in Healthcare” Post‐Graduation Program, Hospital de Amor/Faculdade de Ciências da Saúde “Dr Paulo Prata” (FACISB) Barretos São Paulo Brazil; ^3^ Department of Pediatric Oncology Children's Cancer Hospital, Barretos Cancer Hospital Barretos São Paulo Brazil; ^4^ Barretos Medical School, Faculdade de Ciências da Saúde “Dr Paulo Prata” (FACISB) Barretos São Paulo Brazil; ^5^ Scientific Director, Children's Cancer Hospital Barretos Cancer Hospital Barretos São Paulo Brazil

**Keywords:** bilateral ovarian tumors, case series, exoscope, mature teratoma, ovarian‐sparing surgery, pediatric surgery

## Abstract

**Background:**

Bilateral ovarian tumors in pediatric patients present a surgical challenge requiring complete tumor removal while preserving sufficient ovarian tissue for endocrine function and future fertility. In bilateral disease, preservation is often feasible only on the ovary that retains salvageable parenchyma. Exoscope systems provide high‐definition magnified visualization of the operative field. To our knowledge, this is one of the first reports of exoscope‐assisted ovarian‐sparing surgery in pediatric patients. This study describes the technical feasibility of the technique and the completeness of tumor removal with preservation of ovarian parenchyma.

**Methods:**

We conducted a retrospective case series of four patients with bilateral ovarian tumors who underwent exoscope‐assisted ovarian‐sparing surgery between 2020 and 2024 at a Brazilian pediatric cancer center. Eligible patients had imaging consistent with benign lesions and normal serum tumor markers. Surgical technique combined exoscopic magnification with intraoperative ultrasonography in open procedures.

**Results:**

All patients had mature teratomas confirmed on final pathology. No intraoperative capsular ruptures occurred across the four preservation procedures. Mean follow‐up was 31.0 months (range 20–39). Two patients maintained regular spontaneous menses without hormonal supplementation. One patient developed clinical hypogonadism 8 months after surgery and received hormone replacement therapy, after which regular cycles resumed. One patient was not assessable for spontaneous menstrual recovery because continuous combined hormonal contraception was started after surgery at her request. No tumor recurrence occurred during follow‐up.

**Conclusions:**

Exoscope‐assisted ovarian‐sparing surgery was technically feasible for the contralateral or remaining ovary in adolescents with bilateral mature teratomas. Spontaneous menstrual function was preserved in two of four patients, one required hormone replacement therapy, and one was not assessable. Controlled studies that include objective ovarian reserve markers and extended follow‐up are required before the added value of the exoscope can be established.

## 1 Introduction

Ovarian tumors in pediatric and adolescent patients require surgical approaches that balance oncologic safety with preservation of endocrine function and future fertility. Mature teratomas represent the most common ovarian germ cell tumors in this age group and present bilaterally in approximately 5% of cases at initial diagnosis, with an additional 8.3% of patients developing metachronous contralateral lesions at a median interval of 30.5 months [1]. This bilateral involvement pattern creates a particularly challenging clinical scenario: complete tumor removal from both ovaries must be achieved while preserving sufficient ovarian tissue to maintain hormonal production and reproductive potential [2].

Successful ovarian preservation in bilateral disease depends on precise identification and dissection of tissue planes that separate tumor from healthy parenchyma. The surgical goal is to preserve the maximum amount of cortical tissue containing primordial follicles, as residual ovarian volume correlates with subsequent endocrine and reproductive function [3]. This requirement becomes more demanding in bilateral disease, where cumulative tissue loss across two procedures increases the risk of premature ovarian insufficiency [1, 2].

Current surgical approaches for ovarian preservation in pediatric patients emphasize ovarian‐sparing resection that dissects along the tumor capsule‐parenchyma interface while minimizing trauma to preserved tissue [4]. Preoperative risk stratification using imaging characteristics, tumor markers, and validated scoring systems helps identify appropriate candidates for conservative surgery, and intraoperative frozen section analysis is available as an adjunct to guide the extent of resection in selected cases [2]. Published fertility‐sparing series report favorable menstrual recovery in most patients [3]. When appropriately applied, tissue‐sparing techniques achieve favorable functional results, although their application in bilateral disease is less well described [1, 2].

Exoscope systems represent an evolution in surgical visualization technology, employing external high‐definition cameras to produce magnified video displays that eliminate the need for surgeons to view through microscope oculars. These systems provide 4K or high‐definition stereoscopic 3D imaging with variable optical zoom, mechanical or robotic positioning arms for flexible viewing angles, and real‐time video output that enables team‐wide visualization and procedure recording. Clinical applications across multiple surgical specialties have documented specific advantages: improved surgeon ergonomics with reduced neck and eye strain, enhanced depth perception and magnification for procedures, and team‐wide visualization on monitors for teaching [5]. In oncological surgery, exoscope technology has been applied to sentinel lymph node mapping using near‐infrared fluorescence guidance and documentation for teaching purposes, demonstrating feasibility and practical benefits in pelvic surgical procedures. The ergonomic advantages prove particularly valuable in prolonged cases, where traditional microscope‐bound positioning contributes to surgeon fatigue [5]. To our knowledge, exoscope use for ovarian‐sparing surgery has not been previously reported. The optical magnification of the platform may be applicable to ovarian‐sparing surgery, where identifying the tumor‐parenchyma interface is relevant to tissue preservation.

This case series describes the technical feasibility of exoscope‐assisted ovarian‐sparing surgery for the contralateral or remaining ovary in pediatric patients with bilateral ovarian tumors. The primary objective was to describe the surgical approach and the completeness of tumor removal with preservation of ovarian parenchyma. The secondary objective was to document menstrual function after surgery.

## 2 Methods

We conducted a retrospective case series of patients with bilateral ovarian tumors managed with exoscope‐assisted ovarian‐sparing surgery between 2020 and 2024 at Barretos Cancer Children's Hospital, Barretos, São Paulo, Brazil. This case series has been reported in line with the PROCESS 2025 guideline. The institutional Research Ethics Board approved the study (CAAE protocol #77037617.2.1001.5437/2017). Given the retrospective design with de‐identified data, the requirement for informed consent and parental permission was waived in accordance with institutional policy for minimal‐risk research. The study followed the Declaration of Helsinki and applicable data protection regulations.

Patient selection followed the Childhood and Adolescent Germ Cell Tumor Latin American 2017 protocol (Tumores de Células Germinativas–Grupo de América Latina de Oncología Pediátrica, TCG‐GALOP 2017), a cooperative treatment protocol used across participating Latin American centers [6]. Candidates for ovarian‐sparing surgery had benign imaging features on computed tomography and pelvic magnetic resonance imaging and normal serum alpha‐fetoprotein, beta‐human chorionic gonadotropin, and lactate dehydrogenase. Normal tumor marker values were defined as within institutional reference ranges for age‐appropriate pediatric and adolescent populations. Malignant germ cell tumor and immature teratoma were considered unlikely when imaging showed a well‐circumscribed lesion without solid enhancing components or peritoneal disease and when tumor markers were within age‐specific reference ranges. Every case was discussed at the institutional multidisciplinary tumor board and within the TCG‐GALOP Latin American cooperative group, which reviews ovarian germ cell tumor cases across participating centers.

### 2.1 Surgical Strategy

The choice between resection and preservation for each ovary depended on the amount of salvageable parenchyma, following the TCG‐GALOP 2017 protocol for bilateral ovarian germ cell tumors [6]. The protocol indicates salpingo‐oophorectomy when the ovary has no identifiable salvageable parenchyma, and parenchyma‐sparing resection of the contralateral ovary when salvageable tissue is present. In synchronous cases (Cases 3 and 4), one ovary was extensively replaced by tumor and retained no salvageable parenchyma, even when the lesion appeared small on imaging. Salpingo‐oophorectomy was performed on that ovary. The contralateral ovary retained identifiable parenchyma and underwent magnification‐assisted nodulectomy.

In metachronous cases (Cases 1 and 2), salpingo‐oophorectomy of the index ovary had been performed at a previous operation, a mean of approximately 2 years before the present surgery, and in Case 1 at another institution. The exoscope‐assisted procedure was applied to the metachronous lesion in the remaining contralateral ovary, which developed after treatment of the primary tumor. In all four patients, the magnification‐assisted technique was applied to nodulectomy of the contralateral or remaining ovary, not to both ovaries.

### 2.2 Surgical Technique

**FIGURE 1:**
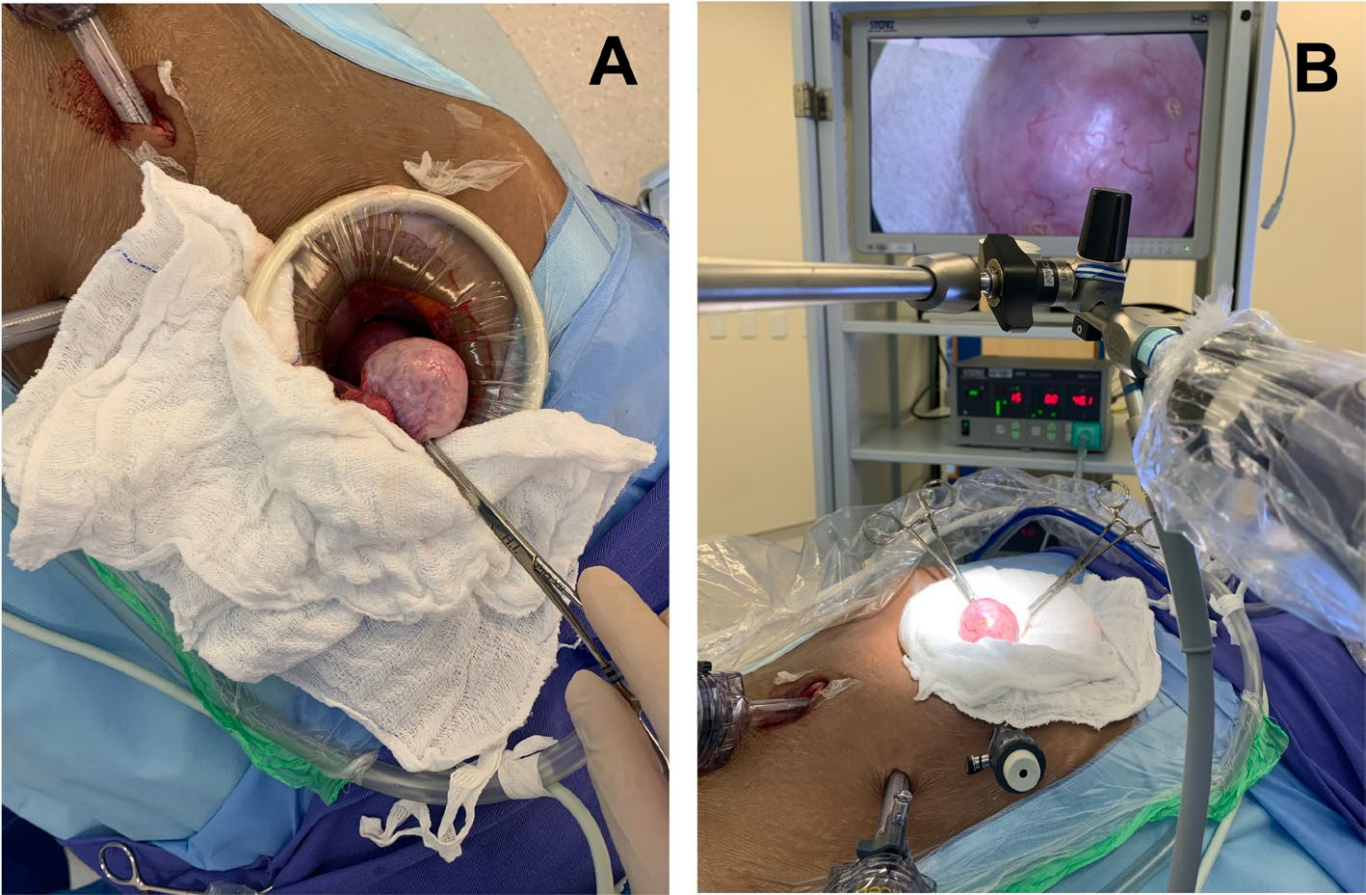
Operative setup for exoscope‐assisted ovarian‐sparing surgery. (A) Exteriorized ovary bearing the ovarian tumor through a Pfannenstiel incision using an Alexis wound retractor, which provides circumferential exposure of the surgical field. (B) Panoramic view of the VITOM 4 K exoscope (Karl Storz) mounted on a positioning arm and directed at the exteriorized ovary. The high‐definition monitor (background) displays the magnified image of the ovarian surface in real time, enabling team‐wide visualization.

All procedures were performed by the same surgical team using a standardized approach. Patients were positioned supine under general anesthesia. A 7–10 cm Pfannenstiel incision provided access to the pelvis. An Alexis wound retractor (Applied Medical, Rancho Santa Margarita, CA, USA) was placed to facilitate exposure, and the affected ovary was exteriorized for examination (Figure 1).

The VITOM 4 K exoscope (Karl Storz, Tuttlingen, Germany) was used as a video‐assisted magnification platform, providing coaxial illumination and an average of 10–15× magnification for dissection, without microvascular anastomosis or microscope‐based reconstruction. The magnified image was displayed on high‐definition monitors visible to the entire surgical team, enabling team‐wide visualization and procedure documentation.

**FIGURE 2:**
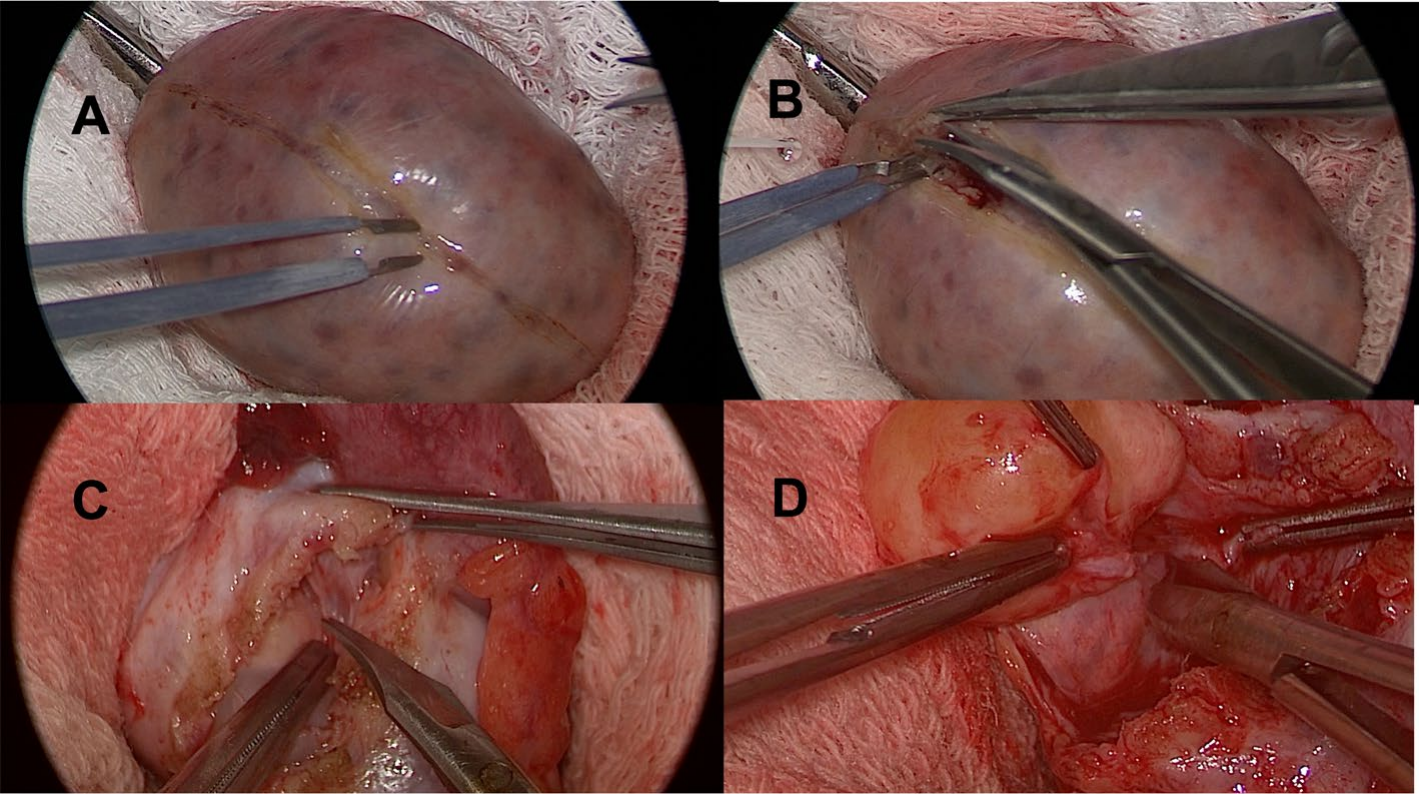
Exoscope‐assisted ovarian‐sparing procedure (VITOM 4 K, 10–15× magnification). (A) Bipolar electrocautery incision of the ovarian cortex. (B) Forceps elevating the ovarian cortex and identifying the cleavage plane between the tumor capsule and normal parenchyma. (C) Progressive dissection of the tumor‐parenchyma interface with visualization of the underlying tumor content. (D) Preserved ovarian parenchyma after complete tumor enucleation, demonstrating intact residual cortical tissue.

Intraoperative ultrasonography with a high‐frequency microtransducer (L8‐18i‐RS linear “hockey‐stick” probe, GE HealthCare, Chicago, IL, USA) delineated the lesion within the ovarian parenchyma and guided the site of the cortical incision. The cortex was incised only after the lesion was identified on ultrasound. Under exoscopic magnification, the capsule‐parenchyma plane was developed and the lesion was enucleated (nodulectomy), with meticulous identification of the tissue plane between the tumor capsule and the normal ovarian cortex. All lesions were well‐circumscribed, without parenchymal invasion, and the residual parenchyma was viable after enucleation. Hemostasis was obtained with focal bipolar electrocautery at the bleeding points, using minimal power settings to limit thermal injury to preserved ovarian tissue (Figure 2). The ovarian bed was not sutured, to avoid parenchymal fibrosis related to suture material. Frozen section was not performed, because the lesions were benign‐appearing and well‐circumscribed, with margins delineated by intraoperative ultrasound.

Intraoperative rupture was defined as visible spillage of tumor content during the procedure. Capsular integrity on pathology referred to whether the capsule of the resected specimen was intact on histopathological examination. A non‐intact capsule on pathology was distinguished from intraoperative rupture, because it may reflect specimen handling or lesion characteristics rather than a surgical event.

### 2.3 Data Collection

Patient demographic and clinical data were extracted from electronic medical records, operative reports, and pathology reports. Collected variables included age at surgery, presentation pattern (synchronous versus metachronous), tumor size and laterality, preoperative imaging and tumor marker results, operative details, completeness of tumor resection (confirmed by histopathology), intraoperative complications including tumor rupture, hemorrhage, or injury to adjacent structures; and histopathological findings. Postoperative menstrual function was assessed through clinical follow‐up. Maintenance of regular menstrual cycles was documented through patient self‐report and clinical examination at follow‐up visits.

Postoperative surveillance followed the TCG‐GALOP 2017 protocol for teratomas [6]. Imaging and serum tumor markers were obtained every 3 months during the first year, every 4 months during the second year, and every 6 months from the third to the fifth year. Imaging modality was ultrasonography or magnetic resonance imaging according to the protocol imaging groups.

Given the small sample size (*n* = 4), analyses were descriptive only. Patient characteristics, tumor measurements, and outcomes are presented as individual case descriptions with summary statistics. Continuous variables are reported as means with ranges where appropriate. No comparative statistical testing was performed.

## 3 Results

Four patients with bilateral ovarian tumors underwent ovarian‐sparing surgery between 2020 and 2024. Exoscope magnification‐assisted dissection enabled identification of the tumor‐parenchyma interface under magnified visualization (Figures 1 and 2). Mean age at surgery was 16.0 years (range 15–17 years). Two patients presented with synchronous bilateral tumors, while two had metachronous contralateral tumors following previous unilateral surgery. Preoperative tumor markers were within normal limits for all patients (Table 1).

**TABLE 1:** Baseline characteristics, operative details, pathology, and postoperative follow‐up (*n* = 4).

	Case 1	Case 2	Case 3	Case 4
Age (years)	17	17	15	15
Follow‐up duration (months)	20	39	35	30
Presentation	Metachronous	Metachronous	Synchronous	Synchronous
Side	Right	Right	Left	Right
Size (cm)	0.7 × 0.5	5.3 × 3.5	4.6 × 3.6 × 3.5	2.2 × 2.5
**Preoperative assessment**				
AFP (ng/mL)	1.2	0.8	2.7	1.5
β‐hCG (mIU/mL)	< 2.39	< 2.39	< 2.39	< 2.39
LDH (U/L)	245	213	230	210
**Surgical Management**				
*Index*				
Procedure	LSO	OSO	OSO	LSO
Intraoperative rupture	No	N/A^a^	No	No
*Contralateral*				
Procedure^g^	Nodulectomy	Nodulectomy	Nodulectomy	Nodulectomy
Intraoperative rupture	No	No	No	No
LOS (days)	1	1	1	1
Surgical complications	No	No	No	No
Reoperation	No	No	No	No
**Pathology**				
*Index*				
Histology	MT	MT	CMT	CMT
Capsule integrity	Yes	N/A^a^	Yes	Yes
*Contralateral*				
Histology	CMT	MT	CMT	CMT
Capsule integrity	Yes	No^f^	Yes	Yes
**Clinical follow‐up**				
Time to first menses post‐op (days)^b^	N/A^c^	455	55	150
Cycle pattern	Amenorrhea^c^	Regular^d^	Regular	Regular
Clinical hypogonadism	No	Yes^e^	No	No
Hormone replacement therapy	No	Yes	No	No

Abbreviations: AFP, alpha‐fetoprotein; β‐hCG, beta‐human chorionic gonadotropin; CMT, Cystic Mature Teratoma; LDH, lactate dehydrogenase; LOS, length of stayLSO, laparoscopic salpingo‐oophorectomy; MT, Mature Teratoma; OSO, open salpingo‐oophorectomy.

^a^ Data unavailable, surgery performed at another institution.

^b^ Time from surgery to resumption or first postoperative menstrual period.

^c^ Continuous combined hormonal contraception started postoperatively at the patient's request.

^d^ Regular cycles following the start of hormone replacement therapy.

^e^ Amenorrhea associated with vasomotor symptoms (hot flashes ± night sweats), diagnosed 8 months after surgery.

^f^ Non‐intact capsule on pathology, without documented intraoperative rupture.

^g^ Index salpingo‐oophorectomy approach: laparoscopic in Cases 1 and 4, open in Cases 2 and 3.

The preservation procedure was nodulectomy in all four patients, completed as planned. No intraoperative tumor rupture or other intraoperative complication occurred (0/4). No significant intraoperative bleeding occurred, and no patient required blood transfusion or reoperation. No patient had ovarian torsion, chemical peritonitis, postoperative adhesions, wound complications, or clinically relevant postoperative pain. Length of stay was 1 day in all four patients.

Pathology showed mature teratoma in all patients (mature teratoma [MT] or cystic mature teratoma [CMT]). Capsule integrity of the preserved ovary was intact in three of four cases. In Case 2, the contralateral specimen had a non‐intact capsule on pathology, without documented intraoperative rupture. The index specimen of Case 2 was resected at another institution, and its data were not available.

Follow‐up duration ranged from 20 to 39 months (mean 31.0). Two patients (Cases 3 and 4) maintained regular spontaneous menstrual cycles without hormonal supplementation throughout follow‐up. Time to first postoperative menses was 55 days in Case 3 and 150 days in Case 4. Case 2 developed clinical hypogonadism 8 months after surgery, with amenorrhea and vasomotor symptoms (hot flashes and night sweats); endocrine assessment showed FSH 130 mIU/mL and estradiol 11.4 pg/mL before hormone replacement therapy. Hormone replacement therapy was started, after which regular cycles resumed, with first menses 455 days after surgery. In Case 3, endocrine assessment showed FSH 2.53 mIU/mL and estradiol 64.8 pg/mL. Case 1 was not assessable for spontaneous menstrual recovery because continuous combined hormonal contraception was started after surgery at the patient's request. No tumor recurrence was detected during follow‐up.

## 4 Discussion

This case series describes the technical feasibility of exoscopic visualization for ovarian parenchyma preservation in adolescents with bilateral mature ovarian teratomas. All four patients underwent tissue‐sparing procedures without intraoperative capsular rupture. Two patients maintained regular spontaneous menses without hormonal supplementation, one required hormone replacement therapy, and one was not assessable. While pediatric literature consistently advocates ovarian‐sparing approaches when oncologically safe [1, 2, 4], specific technical strategies to optimize tissue preservation in bilateral disease remain inadequately described.

The technique combined three elements: exoscopic magnification, direct tactile feedback through open exteriorization, and intraoperative ultrasonography. Magnification supported identification of the capsule‐parenchyma plane, tactile feedback informed the extent of the palpable lesion, and ultrasonography delineated the lesion and guided the cortical incision. Because these elements were applied together and no control group was available, the design does not allow the contribution of any single element, including the exoscope, to be isolated. The reported outcomes reflect the combined technique rather than the exoscope alone.

Single‐port robot‐assisted laparoscopic surgery has been reported as a feasible alternative for resection of pediatric benign ovarian tumors [7]. Robotic platforms offer magnified three‐dimensional visualization and articulated instruments, although high cost and limited availability restrict their use. The exoscope provides magnified visualization at lower cost and integrates with open exteriorized surgery and intraoperative ultrasonography.

Reported spillage rates in laparoscopic management of pediatric MTs range from 30% in single‐center series to 52% in multicenter cohorts [8]. These figures are not comparable to the present series. Our four patients had benign‐appearing, well‐circumscribed lesions selected for open exteriorized surgery with intraoperative ultrasonography, whereas the laparoscopic series include different tumor sizes, surgical approaches, and definitions of rupture. The absence of intraoperative rupture in four selected open procedures does not indicate superiority of the exoscope or of the open approach over laparoscopy.

Beyond technical execution, the oncologic adequacy of ovarian‐sparing approaches depends on both complete tumor removal and long‐term disease control. No tumor recurrence was observed during the follow‐up period, consistent with expected outcomes for completely resected MTs. Capsular integrity was intact in three cases; in Case 2 the contralateral specimen had a non‐intact capsule on pathology, without documented intraoperative rupture. Pediatric series report recurrence rates following ovarian‐sparing surgery for MTs ranging from 2% to 8.6%, with most recurrences occurring within the first 2–5 years after initial surgery [8, 9]. A national pediatric cohort documented second events, including ipsilateral recurrences and contralateral new tumors, in a comparable proportion of patients, at a median of approximately 30 months [1]. Individual series have reported recurrences as late as 74 months postoperatively [8], emphasizing the necessity of extended surveillance beyond the immediate postoperative period. Intact capsular integrity is regarded as a favorable prognostic indicator, as capsular rupture has been associated with increased risk of peritoneal implantation and recurrence in some series [1]. However, the clinical significance of intraoperative spillage specifically in benign MTs remains debated, with several studies reporting no chemical peritonitis or increased recurrence after contained spillage in laparoscopic approaches [10].

While oncologic safety represents one dimension of successful tissue‐sparing surgery, preservation of ovarian endocrine function constitutes the ultimate goal. Spontaneous menstrual function without hormonal supplementation was documented in two of four patients. Large pediatric series report menstrual recovery exceeding 90% after ovarian‐sparing surgery [3, 8, 10], but these series predominantly describe unilateral procedures, and data after bilateral ovarian‐sparing surgery in adolescents are limited.

The development of hypogonadism in Case 2 merits consideration of multiple potential contributing factors. This patient underwent metachronous bilateral surgery, with initial salpingo‐oophorectomy performed at another institution. Cumulative surgical trauma from repeated ovarian interventions has been associated with compromised ovarian reserve in broader gynecologic literature [3], though quantitative pediatric data isolating this effect are lacking. Objective ovarian reserve was not systematically assessed. Anti‐Müllerian hormone was not measured before or after surgery, and antral follicle count and Doppler perfusion were not obtained. FSH and estradiol were available in two patients. Menstrual history alone is an inadequate surrogate for ovarian endocrine and reproductive function, and future tissue‐sparing protocols should incorporate baseline and serial AMH measurement, antral follicle count, and ovarian volume.

Several limitations warrant acknowledgment, with the most critical being the small sample size, positioning these findings as hypothesis‐generating rather than definitive. The retrospective design with procedures performed by a single surgical team limits assessment of technique reproducibility and introduces potential selection bias; consequently, observed outcomes may not generalize to other surgeons or practice settings. Data from Case 2's initial surgery, performed at an external institution, are incomplete, precluding full analysis of factors contributing to subsequent hypogonadism. The absence of a comparison group receiving conventional, loupe‐assisted, or laparoscopic surgery prevents direct assessment of exoscope‐added value; observed outcomes may reflect surgical expertise, favorable case selection, or multimodal technique integration rather than magnification per se. Reproductive outcomes, the ultimate endpoint of fertility‐preserving surgery, cannot be evaluated given the patients' ages and follow‐up timeframe. Natural conception attempts typically occur years after surgery in this population, necessitating extended surveillance to capture long‐term reproductive success.

In this small, selected case series, exoscope‐assisted ovarian‐sparing surgery of the contralateral or remaining ovary was technically feasible, with no intraoperative rupture and no short‐term recurrence. The added value of the exoscope over conventional open ovarian‐sparing surgery, loupe‐assisted surgery, or laparoscopy was not established, because the study had four patients and no control group. Cost, equipment availability, and training currently limit generalizability to specialized referral centers. Validation of novel surgical techniques in pediatric populations requires iterative evidence generation: proof‐of‐concept case series, followed by comparative cohort studies, and ultimately prospective trials when equipoise exists. Comparative studies with objective ovarian reserve assessment, operative metrics, and longer follow‐up are needed before this approach can be recommended for routine use.

## Author Contributions

Conceptualization: R.C.R. and W.E.O. Data curation: B.R.R., G.E.M., and L.S.S. Investigation: W.E.O., B.R.R., G.E.M., G.S.O., L.S.S., and A.L.B.N. Methodology: R.C.R., W.E.O., and L.F.L. Project administration: W.E.O. and R.C.R. Supervision: R.C.R. and L.F.L. Validation: B.R.R., R.C.R., and W.E.O. Visualization: L.S.S., A.L.B.N., and R.C.R. Writing – original draft: W.E.O., A.L.B.N., and B.R.R. Writing – review and editing: W.E.O., B.R.R., R.C.R. All authors reviewed the manuscript.

## Funding

The Article Processing Charge (APC) for the publication of this research was funded by the Coordenação de Aperfeiçoamento de Pessoal de Nível Superior—Brasil (CAPES) (ROR identifier:00x0ma614).

## Disclosure

During the preparation of this work the authors used Grammarly, an AI‐assisted writing tool, in order to check grammar and improve text cohesion. After using this tool, the authors reviewed and edited the content as needed and take full responsibility for the content of the published article.

## Consent

No animal subjects were used in this study. All human subjects are published and follow ethical standards.

## Conflicts of Interest

The authors declare no conflicts of interest.

## Data Availability

The data that support the findings of this study are available on request from the corresponding author. The data are not publicly available due to privacy or ethical restrictions.
